# Optimization of culture parameters for α-glucosidase production from suspension culture of moss *Hyophilla nymaniana* (Fleish.) Menzel

**DOI:** 10.1186/s43141-020-00098-8

**Published:** 2020-12-14

**Authors:** Rashmi Mishra, Ramesh Chandra

**Affiliations:** 1 Department of Bio-Engineering Birla Institute of Technology Mesra, Ranchi, Jharkhand 835215 India; 2 ICAR-Indian Institute of Natural Resins and Gums Namkum, Ranchi, Jharkhand 834010 India

**Keywords:** Taguchi method, *Hyophilla nymaniana* (Fleish.) Menzel, Analysis of variance, Alpha glucosidase, Orthogonal array

## Abstract

**Abstract:**

**Background:**

Bryophytes, comprising of the second largest group in the plant kingdom, has attracted a great deal of attention in recent years due to its immense potential to produce biopharmaceuticals. But studies conducted to better understand their chemical composition are limited and scattered. In the present investigation, sequential optimization strategy, based on statistical experimental designs, was employed to enhance the production of α-glucosidase enzyme from moss *Hyophilla nymaniana* (Fleish.) Menzel by using Taguchi methodology. L16 orthogonal array and five physical parameters including sugar, temperature, pH, rpm, nitrogen source were considered as key parameters for enzyme production. The optimal level of each parameter for maximum glucosidase production by the moss was determined. Analysis of variance (ANOVA) was performed to evaluate statistically significant process factors.

**Results:**

Based on statistical analysis (ANOVA), the optimal combinations of the major constituents of media for maximal α-glucosidase production were evaluated as follows: dextrose 2% contributed maximum on α-glucosidase production followed by ammonium nitrate 1.5%, temperature 24 °C, pH 5.6, and RPM 120. Predicted results showed an enhanced glucosidase (53%) than the basal production medium.

**Conclusion:**

The present study highlighted that Taguchi design of experiments approach is better than the conventional optimization technique to determine the optimum level of each of the significant parameters that brings maximum enzyme production.

## Background

α-glucosidases (EC 3.2.1.20, α-d-glucoside glucohydrolase) is an exoglycosidase that catalyze the release of α-D-glucose from the non-reducing end α-glucosides or from complex polymers with α-(1-4) bonds, such as malto-oligosaccharides, soluble starch, amylose, and glycogen, although it cannot act on raw starch [1, 2]. Besides α-glucosidases, oligo-1, 6-glucosidase, and sucrase-isomaltase are also categorized as α-glucosidases. This α-glucosidases is ubiquitously present in nature and are classified into three categories. Type I glucosidase prefers aryl glucosides and sucrose, more efficiently than maltose, type II prefers maltose and isomaltose and has low activity toward aryl glycosides while type III has the same specificity as type II but also attack starch [3, 4]. Plants, α-glucosidase are ubiquitous in nature and have many important functions. Apparently, the main function of these enzymes consists in the hydrolysis of oligosaccharides produced from starch to yield glucose [4] which serves as an energy source for the developing plant. Apart from that plant α-glucosidase could initiate the degradation of natural starch granules in pea chloroplasts and barley seeds in the absence of α-amylases [5]. Furthermore, two α-glucosidases I and II isolated from mung bean have been reported to play a key role in the biosynthetic processing of asparagine-linked oligosaccharides [6, 7]. Besides these facts, a large number of α-glucosidase inhibitors isolated from the plant are being used as anti-diabetic drugs for diabetes mellitus type II [8]. Furthermore, α-glucosidase deficiency is directly related to rare multisystem genetic disorder Pompe disease that is characterized by impaired or deficiency of the lysosomal α-glucosidase. Apart from this, plant α-glucosidase is extensively used in biotechnology and has important applications in both the food and the pharmaceutical industries. Some α-glucosidases have transglucosylase activity and produce isomalto- and malto-oligosaccharides (IMOs) such as isomaltose, maltotriose, panose, and nigerose [9, 10]. Therefore, α-glucosidases could be used to produce IMOs with prebiotic activity [4, 11], glucose from starchy sources in industrial brewing, also for syrup and biofuel production and recently in medical biosensors [12]. In view of the involvement of the enzyme in various applications, its higher production is desirable. Glucosidase has wide occurrence in nature and has been well studied in bacteria, fungi, animals, and plant. However, α-glcosidase from lower plant-based production system especially from suspension culture of mosses has been infrequently studied. Till now, only a little work has been done in cultivating mosses in suspension culture in order to make them available in bulk amount for their potential use or further bioprospecting studies. The main advantage of the moss suspension cultures in biopharmaceuticals applications is the fact that they have similarity in protein N-glycosylation pattern with the higher plants and possess an exceptionally high rate of homologous recombination in mitotic cells. Thus, making it highly preferred system for humanization of glycosylation pattern through homologous recombination [13, 14]. Furthermore, suspension culture of mosses can be an ideal material for morphogenetic, genetic, physiological, biochemical, and molecular studies [15]. Although, it has been reported that establishment of plant tissues and organs under axenic conditions was first attempted and profitably employed in bryophytes, especially mosses, very little is known about its in vitro culture. In the present study, we endeavor to establish a generalized protocol for the standardization of α-glucosidase production from the suspension culture of moss *Hyophilla nymaniana*.

Process parameter like media composition and the different variables of media interacting among each other were optimized. The conventional optimization procedures involves one variable at a time (OVAT) design, require time consuming experimental work and cannot provide information about the mutual interactions of the parameters [16, 17]. On the contrary, statistical design of experiments helps to investigate the influence of controlled factor in multivariate system.

Recent literature reviews reveal that application of Taguchi methodology to optimize reaction variables in biochemical processes. Taguchi orthogonal array (OA) design of experiment (DOE) involves the study of any given system by a set of independent variables (factors) over specific levels of interest [18–20]. It involves the use of predefined orthogonal arrays to study a maximal number of factors at selected levels with a minimal set of experiments. It allows us to study the influence of individual factors, to establish the interaction between them, and finally to calculate performance at the optimum levels obtained [21–23].

To our knowledge, no investigation has been reported to describe the sequential optimization strategy for α-glucosidase enzyme production from moss suspension culture. In the present work, we report for the first time sequential optimization strategy for α-glucosidase production Taguchi design of experiments.

## Methods

### Plant material

Healthy mature undehicense capsules were collected from different parts of Ranchi, Jharkhand, India. Taxonomic identity was confirmed by comparing with authenticated herbarium specimen at Botanical Survey of India, Kolkata. A voucher specimen of the plant was kept in the herbarium of the institute (KC-002).

### Culture initiation

The healthy mature undehicense capsules of *H. nymaniana* were disinfected with 2% freshly prepared sodium hypochlorite solution for 5 min, washed with 5 × 1 ml sterile distilled water, and cultured in hormone-free MS medium at 25 °C and with photon flux density of 18 lmol/m^2^/s with 16:8-h light/dark photoperiod. The young protonemal cells developed were taken for the initiation of suspension culture.

### Cultivation of the plant in suspension culture

To establish suspension cultures, 4 to 6 weeks old actively growing protonemal cells were first transferred into the liquid MS medium (mg/L) that contains NH_4_NO_3_, 1650; KNO_3_, 1900; CaCl_2_.2H_2_O, 440; MgSO_4_.7H_2_O, 370; KH_2_PO_4_, 216; KI, 0.83; H_3_BO_3_, 6.2; MnSO_4._4H_2_O, 22.3; ZnSO4.7H_2_O, 8.6; Na_2_Mo–O_4_.2H_2_O, 0.5; CuSO_4_.5H_2_O, 0.05; CoCl_2_.6H_2_O, 0.05; FeSO_4_.7H_2_O, 26; Na_2_EDTA.2H_2_O, 37.3; myo-inositol, 90; nicotinic acid, 1; pyridoxine HCl, 0.5; thiamine-HCl, 1; biotin, 0.01; glucose, 20,000. (Cells were sub-cultured every 3 weeks at 25 °C and with photon flux density of 18 lmol/m^2^/s with 16:8-h light/dark photoperiod. Fine cell clumps of average diameter ca. 0.5 mm harvested during exponential growth phase were used as inoculums. Fast-growing cells were suspended in 50 mL medium in 250 mL conical flasks and were cultured on a rotary shaker (100 rev/min) at 25 °C under illumination by fluorescent lamps using a 16:8-h light/dark photo-period. Culture experiments were carried out in triplicates. Data were denoted as mean ± SD.

### Extraction of crude enzyme

The cell in the suspension culture routinely monitored for the determination of the biomass. The inoculated flasks were incubated for different time intervals (7, 14, 21 28, 35, and 42 days) and checked for biomass production. Crude enzyme extraction (28-day-old culture) carried out from the suspension culture by centrifugation at 10,000×*g* for 10 min at 4 °C. The cells were resuspended in phosphate buffer containing 10 mM beta mercaptoethanol and subjected to sonication at an amplitude of 35% 15,000 J energy and a pulse rate of 10 s on and 10 s off for 10 min at 4 °C. The cell-free supernatant was used as crude enzyme to perform enzyme assay.

### Enzyme assay and protein estimation

The α-glucosidase activity was recorded as per the method by Constantino et al. 1990 with some modifications. One hundred microliters of enzyme solutions was added to 200 μl (10 mM) of substrate (*p*-nitrophenyl-α-D-glucopyranoside) dissolved in 200 μl citrate buffers (100 mM, pH 4.5). The assay mixtures were incubated for 30 min, and the reaction was terminated by the addition of 1 ml sodium carbonate (0.1 M, pH 9.5). Liberated *p*-nitrophenol was measured spectrophotometerically at 405 nm. One unit of enzyme activity was defined as the amount of enzyme hydrolyzing 1 μM of substrate per minute. Protein content was estimated by the dye-binding method of Bradford et al. by using bovine serum albumin (BSA) as a standard [24].

### Taguchi methodology

One-factor-at-a-time and Taguchi methods were used for optimization of culture condition. The optimum incubation period was determined in a separate experiment, by one-variable-at-a-time method. The inoculated flasks were incubated for different time intervals (7, 14, 21, 28, 35, and 42 days). Initial screening of the culture parameters affecting enzyme production was conducted by OFAT method; later, all factors were optimized that control the enzyme production process by Taguchi DOE method. Five typical fermentation factors, i.e., carbon source (dextrose), nitrogen source (NH_4_NO_3_), pH, temperature, and agitation which have a significant influence on enzymes production were considered by performing OVAT experiments (data not shown), and the chosen parameters were varied at four different levels in Taguchi design of experiments (Table 1). The selected variables were arranged into an orthogonal array (L16 orthogonal array for the representative experiments) (Table 2). With respect to medium optimization, each column would correspond to individual medium components, and each row would represent trails with different combinations of factors. Each of the above-selected components were taken at four defined concentrations, covering the range over which its effect can be determined. Once the optimization procedure was completed, Qualitek-4 software (version 17.1.0) automatic design of Taguchi experiments was used to process the experimental data. “Bigger-is-better” quality characteristics were adopted for determining the optimum culture conditions for enhancing the production of enzymes. This is also used to identify the influence of specific factors on enzymes production. With the help of software, the optimum conditions were determined and production of enzymes was predicted at these conditions.

**Table 1:** Selected process parameters and respective levels in the experimental design

Sl. no.	Factors	Level 1	Level 2	Level 3	Level 4
1	Dextrose % (w/v)	1	1.5	2	3
2	NH_4_NO_3_ % (w/v)	0.5	1	1.5	2
3	Temperature (^°^C)	22	24	26	28
4	pH	5.4	5.6	5.8	6.0
5	Agitation (rpm)	80	100	120	140

**Table 2:** L-16 Orthogonal array of Taguchi experimental design for α-glucosidase production from protonemal biomass of *H. nymaniana*

Run	Dextrose	NH_**4**_NO_**3**_	Temperature	pH	Agitation	α-glucosidase (U/ml)
1	L1	L1	L1	L1	L1	7.8
2	L1	L2	L2	L2	L2	9.2
3	L1	L3	L3	L3	L3	10.9
4	L1	L4	L4	L4	L4	8.5
5	L2	L1	L2	L3	L4	9.2
6	L2	L2	L1	L4	L3	8.5
7	L2	L3	L4	L1	L2	11.5
8	L2	L4	L3	L2	L1	4.5
9	L3	L1	L3	L4	L1	10.2
10	L3	L2	L4	L3	L2	9.5
11	L3	L3	L1	L2	L4	11.8
12	L3	L4	L2	L1	L3	12.5
13	L4	L1	L3	L2	L3	7.9
14	L4	L2	L3	L1	L4	9.4
15	L4	L3	L2	L4	L1	9.2
16	L4	L4	L1	L3	L2	7.8

### Statistical analysis and validation of experiments

Protonemal biomass and α-glucosidase production values were presented as mean ± standard deviation from five experimental data sets. To further validate the methodology, the obtained experimental data was processed in the Qualitek-4 software with bigger-is-better quality characteristics for the determination of the optimum culture conditions for α-glucosidase production, to identify the influence of individual factors on the enzyme production and to estimate the performance at the optimum conditions.

## Effective factors and levels

### Results

The optimizations of various process parameters for α-glucosidase production by sequential optimization approaches were attempted and were applied in the present part of the study. The first approach deals with the determination of the optimum incubation period, by one-variable-at-a-time method. The second approach deals with screening for nutritional factors affecting the glucosidase production in moss suspension culture. The third approach is to optimize the factors that control the enzyme production process.

### Determination of the optimum incubation period

**Fig. 1:**
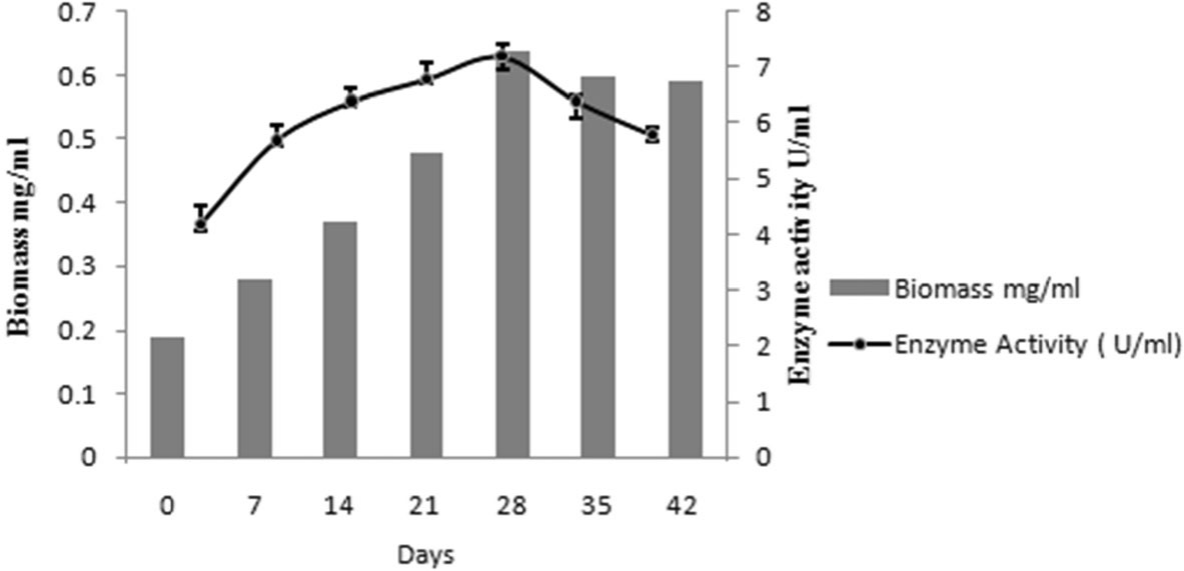
Effect of protonemal growth on α-glucosidase production in *H. nymaniana.* Error bars represents mean ± standard deviation from five experimental data sets

One variable-at-a-time method is used to determine the optimum incubation period for glucosidase production. Results indicated that at different incubation periods 7, 14, 21, 28, 35, and 42 days the activity was 4.2, 5.7, 6.4, 6.8, 7.2, 6.4, and 5.8, respectively. The incubation period for 28 days was the most favorable for maximum glucosidase production where the activity increased about 1.2-fold compared to that at 7-day-old culture (Fig. 1).

### Evaluation of the process parameters affecting glucosidase productivity

**Fig. 2:**
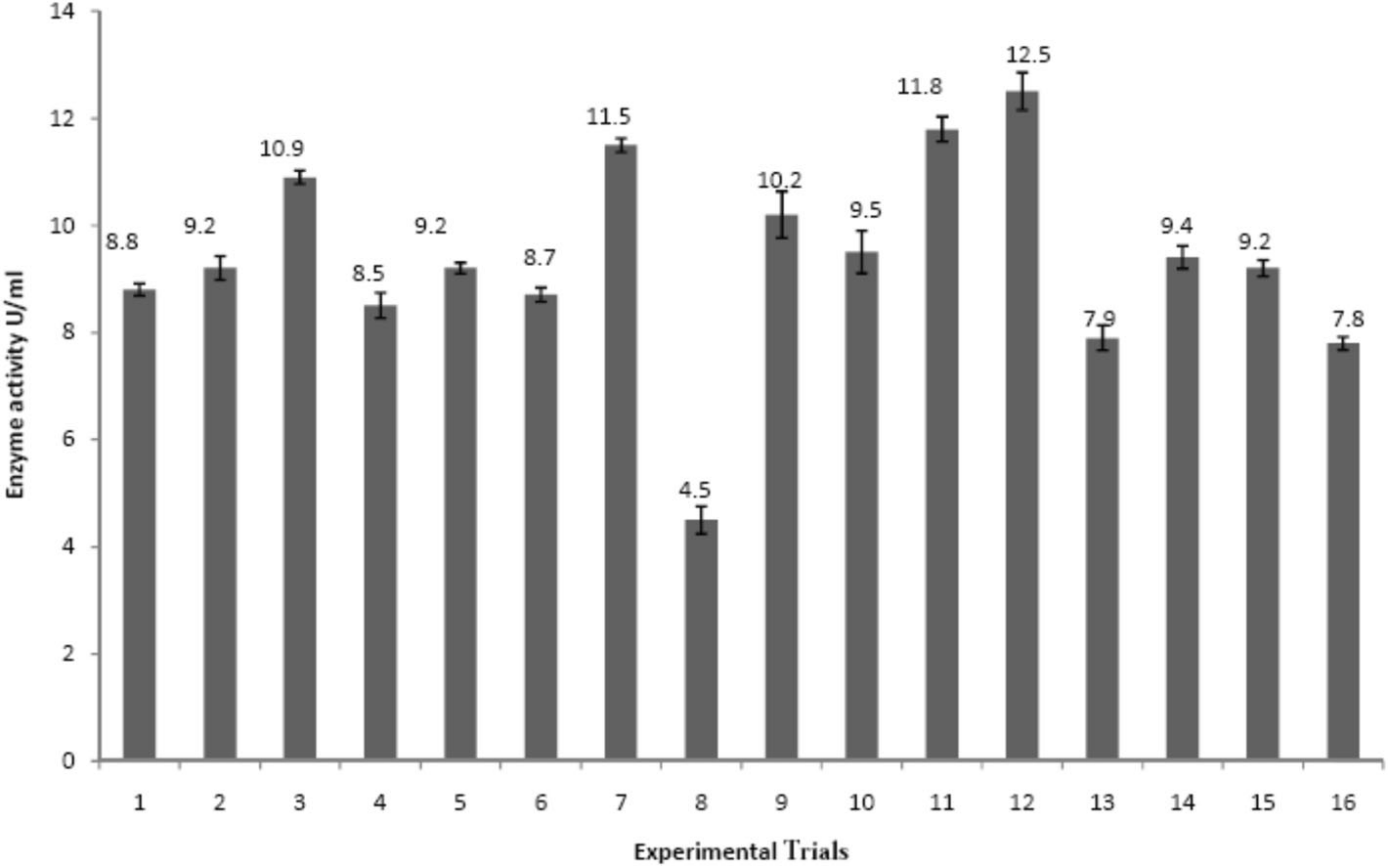
Enzyme activity on 16 trial conditions

Sixteen different trial conditions were used for the analysis of suspension culture parameters and identifying optimum levels of proposed factors (Fig. 2). The averages of glucosidase activity for the different trials (experimental) together with the predicted activity are shown in Table 2. Optimization was done on the basis of the results of these 16 experimental trials. Moreover, after optimizing all these production parameters in 16 trial conditions, it has been analyzed that different factors affect the enzyme production at different levels. The favorable levels of different factors can be evaluated from the value of Severity Index (SI). The SI value gives idea about the interaction between two factors which may help us in understanding of overall process of analysis. The SI value of 100% indicates a 90° angle between the lines versus 0% SI for parallel lines is given in Table 3.

**Table 3:** The estimated interaction Severity Index of the factors under study

Sl. no.	Interacting factor pairs (order based on SI)	Columns	Severity Index (SI%)	Col	Optimum
**1**	Dextrose × pH	1 × 4	51.83	5	[3,1]
**2**	Temperature × pH	3 × 4	41.90	7	[2,1]
**3**	Dextrose × RPM	1 × 5	30.44	4	[3,3]
**4**	Dextrose × NH_4_NO_3_	1 × 2	15.09	3	[3,4]
**5**	NH_4_NO_3_ × RPM	2 × 5	13.38	7	[4,3]
**6**	pH × RPM	4 × 5	10.57	1	[1,3]
**7**	Dextrose × temperature	1 × 3	6.29	2	[3,2]
**8**	Temperature × RPM	3 × 5	2.96	6	[2,3]
**9**	NH_4_NO_3_ × temperature	2 × 3	1.03	1	[4,2]
**10**	NH_4_NO_3_ × pH	2 × 4	0.12	6	[4,1]

After the consideration of SI, it has been observed that the pH and dextrose have maximum effect on α-glucosidase production while pH and NH_4_NO_3_ have the minimum. The rest of the factors have intermediate effect on enzyme production. Different factors at their best levels and their respective contributions are mentioned in Table 4.

**Table 4:** Different factors at their best levels and their respective contributions

Sl. no.	Factors	Level description	Optimum level	Contribution
1	Dextose	2	3	1.707
2	NH_4_NO_3_	1.5	3	1.535
3	pH	5.6	2	0.955
4	Temperature	24 ° C	2	1.112
5	RPM	120	3	0.636
Total contribution from all factors				5.953
Current grand average of performance				9.203
Expected result at optimum condition				13.446

### Optimum conditions and validation of experiments based on ANOVA

**Fig. 3:**
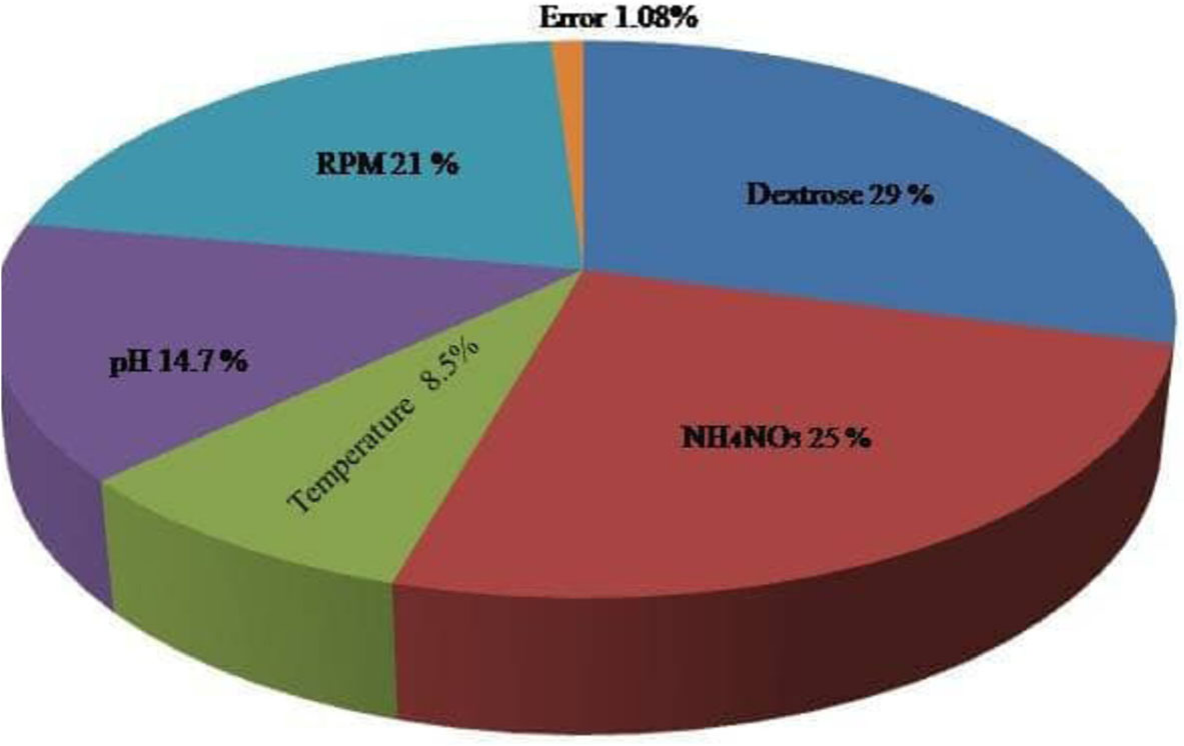
Percentage of various factors contributing to overall performances

**Fig. 4:**
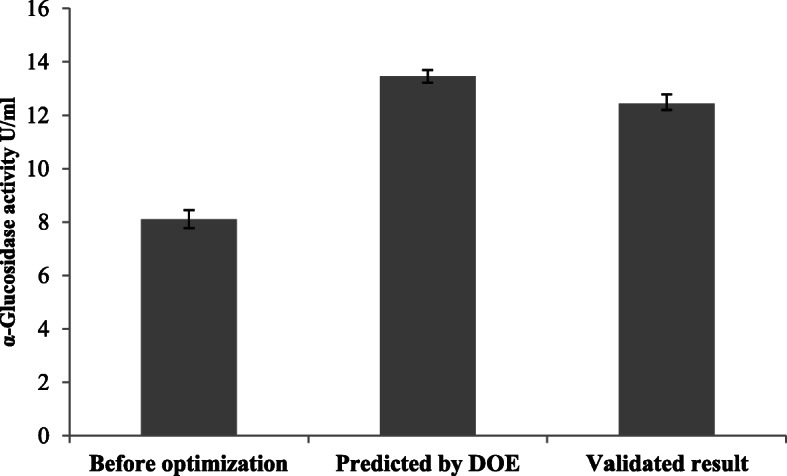
Comparison of enzyme activity before and after optimization process. Error bars represents mean ± standard deviation from five experimental data sets

Analysis of variance (ANOVA) was used to analyze the experimental data and to determine the variation of the result due to each factor. ANOVA reports along with the percentage of contribution of each factor are shown in Table 5. Results indicated that dextrose contributed the maximum impact (29.27%) on α-glucosidase activity followed by NH_4_NO_3_ (25.15%), RPM (21.21%), pH (14.74 %), and temperature (8.528%). The significant contribution of each factor in glucosidase production is depicted in Fig. 3. Individually, each factor influenced the enzyme activity at a certain level. However, this significant factor gives maximum yield when these factors act collectively, which may be due to the interactive effect of different factors. Statistical calculations predicted that, if the conditions were chosen as shown in Table 4, the enzyme production should reach 13.44 U/mL. However, after performing the experiment at the suggested condition, the produced glucosidase was about 12.43 U/mL, but since the difference between predicted and actual result was only about 1.01%, it should be regarded as acceptable. Therefore, comparing with 8.1 U/mL, produced before, a further increase of about 1.5-fold was achieved after optimization of the culture parameters through Taguchi DOE method (Fig. 4). This reflects the necessity and value of optimization process. Number of studies reports the optimization of process parameters for the production of enzyme from different sources [25–27] but to our knowledge, no investigation has been reported to describe the sequential optimization process of the various factors for the enzymes production from the moss *H. nymaniana*.

**Table 5:** Analysis of variance of the factors in step 1 using average of results

S. no.	Factors	DOF (f)	Sum of square	Variance	***F*** ratio	Pure sum	Percent ***P***%
1	Dextrose	3	34.111	11.37	279.693	33.989	29.278
2	Ammonium nitrate	3	29.306	9.768	240.298	29.184	25.14
3	Temperature	3	10.019	3.339	82.155	9.897	8.528
4	pH	3	17.25	5.750	141.446	17.128	14.754
5	RPM	3	24.749	8.249	202.933	24.627	21.214
**Error**		16	0.65	0.04			1.089
**Total**		31	231.734				100%

## Discussion

Extensive research has demonstrated that the production of recombinant biopharmaceuticals is a strongly growing area in the pharmaceutical industry. While most products to date are produced in mammalian cell cultures, plant-based production systems are gaining importance over the last years. Today different plant systems are being explored to successfully grow them in axenic culture conditions in order to meet the demand of biopharmaceutical industry. Although attempts are being made for the successful establishment of suspension culture from different bryophytes species, very few have been attempted for the establishment of cell suspension culture from mosses. Some of the moss species that have been reported for the establishment of suspension culture includes *Atrichum undulatu* [28] peat moss *Sphagnum* imbrications [29]. Recently, establishment of suspension culture system in mosses have gain momentum with the successful establishment of in vitro and suspension culture of the moss model plant *Physcomitrella patents*. Several researches have established bioreactor-based suspension culture for a moss *P. patens* for the production of biopharmaceuticals [15, 30, 31]. In the present study, an attempt has been made for the establishment of the suspension culture of the moss *H. nymaniana* by optimizing the culture parameters to increase the production of glucosidase enzyme by Taguchi DOE method. Although, a number of studies reports the optimization of process parameters by statistical method for the production of enzyme from different sources [30, 31 32], but this is our first report that describes a complete method of production and media optimization of α-glucosidase in hormone-free MS medium by statistical method which has not yet been studied in lower plants mosses.

## Conclusions

α-glucosidases are an important N-glycosylation enzymes used in pharmaceutical industry; therefore, its production in large scale is of paramount importance. Till now, various literatures convey the production of α-glucosidase from different animal and plant sources, but no report for the production of enzyme from moss suspension culture was reported till date. This is the first report on optimization of culture condition for α-glucosidase production by Taguchi methodology. Optimized media with (29.27%) dextrose contributed the maximum impact on α-glucosidase activity followed by NH_4_NO_3_ (25.15%), RPM (21.21%), pH (14.74%), and temperature (8.528%). Maximum glucosidase production of 12.43 U/ml was achieved under the optimal experimental conditions. Before optimization, the enzyme production was 8.1 U/ml but an increase of 1.5-fold was achieved after optimization of the culture parameters through Taguchi DOE method.

## Data Availability

The datasets used and/or analyzed during the current study available from the corresponding author on reasonable request.

## Abbreviations

ANOVA: Analysis of variance
DOE: Design of experiment
NH_4_NO_3_: Ammonium nitrate
pH: Potential hydrogen
RPM: Rotation per minute
SI: Severity Index
